# Resolution of Breast Cancer in a Patient With Thyroid Stimulating Hormone-Secreting Pituitary Neuroendocrine Tumor With the Combination of Chemotherapy and Lanreotide

**DOI:** 10.7759/cureus.66221

**Published:** 2024-08-05

**Authors:** Saki Komai, Nozomi Harai, Ippei Tahara, Yuko Nakayama, Kyoichiro Tsuchiya

**Affiliations:** 1 Diabetes and Endocrinology, University of Yamanashi Hospital, Chuo, JPN; 2 Pathology, University of Yamanashi Hospital, Chuo, JPN; 3 First Department of Surgery, University of Yamanashi Hospital, Chuo, JPN

**Keywords:** trastuzumab deruxtecan therapy, lanreotide, igf-1r, sstr2, breast cancer, tsh-pitnet

## Abstract

Thyroid stimulating hormone-secreting pituitary neuroendocrine tumor (TSH-PitNET) is a rare disease in which pituitary adenomas secrete excessive amounts of TSH, and TSH is not suppressed despite high blood levels of thyroid hormone. Somatostatin analogs (SSAs) like lanreotide are used to control TSH secretion and manage symptoms in cases where surgery is not fully effective or feasible. The treatment of choice for human epidermal growth factor 2 receptor (HER2)-positive metastatic breast cancer is generally chemotherapy and anti-HER2 therapy. A 52-year-old woman was diagnosed with Graves’ disease 26 years ago and stopped going to the hospital after several years of treatment with thiamazole. She had a right breast mass two years prior and visited the Department of Breast and Endocrine Surgery in our hospital one year prior, where she was diagnosed with T3N3M1, stage 4 breast cancer with a mass 52 mm in diameter in the right breast and metastasis in the 12th thoracic vertebra. Breast cancer receptor status was negative for the estrogen receptor, negative for the progesterone receptor, and positive for HER2. She was also found to have an enlarged thyroid gland, palpitations, inappropriate TSH secretion, and a 6 mm nodule on the pituitary gland, which was diagnosed as a TSH-PitNET. She was treated for breast cancer with trastuzumab deruxtecan therapy and for TSH-PitNET with lanreotide. One month after starting lanreotide, pituitary, and thyroid function improved to normal, and four months later, the breast mass was significantly reduced to 16 mm in diameter and a mastectomy was performed. The size of the pituitary adenoma remained unchanged during observation. Remarkably, the mastectomy specimen was free of cancer cells and showed a pathologically complete response. Needle biopsy specimens at the time of breast cancer diagnosis were positive for somatostatin receptor 2 (SSTR2) and insulin-like growth factor 1 receptor (IGF-1R) immunostaining. However, both were negative in the mastectomy specimen. Recently, SSTR2 and IGF-1R were reported to be expressed in breast cancer, and several clinical trials of SSAs for breast cancer have been conducted. SSAs are effective in improving pituitary and thyroid functions against TSH-PiTNET, and in combination with chemotherapy, they may have synergistic antitumor effects in patients with SSTR2-positive breast cancer.

## Introduction

Excessive thyroid hormone levels increase the estrogen/androgen ratio and the incidence of breast cancer [1,2]. However, to date, there have been no reports of breast cancer complications in thyroid-stimulating hormone-secreting pituitary neuroendocrine tumors (TSH-PitNETs).

A TSH-PitNET is treated with surgery as the first-line therapy; however, somatostatin analogs (SSAs) are a treatment option for the management of pituitary and thyroid functions [3]. Trastuzumab deruxtecan therapy (T-DXd) is an option for chemotherapy in patients with human epidermal growth factor receptor 2 (HER2)-positive metastatic breast cancer [4]. Recently, somatostatin receptor 2 (SSTR2) and insulin-like growth factor 1 receptor (IGF-1R) have been reported to be expressed in breast cancer [5-8], and several clinical trials of SSAs in breast cancer have been conducted. However, its efficacy has not been consistent, and it is not a standard treatment [5,9,10].

Herein, we report a case of TSH-PitNET combined with breast cancer treated with T-DXd and lanreotide, which showed marked shrinkage of the breast mass, resolution of cancer cells, and normalization of pituitary and thyroid functions.

## Case presentation

**Figure 1:**
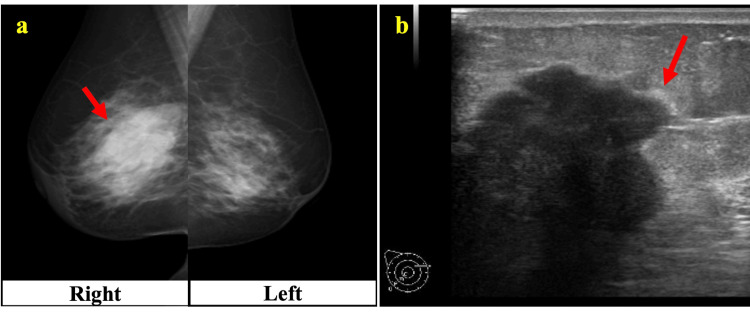
Imaging findings of breast cancer a: Mammography, the left side shows the right breast, and the right side shows the left breast. An irregularly shaped mass with spiculated margins is seen (arrow). b: Breast ultrasonography, an irregularly shaped mass is seen (arrow).

A 52-year-old woman was diagnosed with Graves’ disease 26 years prior and treated with thiamazole; however, her thyroid function did not improve, and she stopped visiting the hospital after several years. Twelve years ago, she complained of palpitations and visited the University of Yamanashi Hospital. She was diagnosed with inappropriate secretion of TSH and pituitary adenoma, leading to the suspicion of TSH-PitNET. A beta-blocker was initiated, but she stopped visiting the hospital again after a few years. Two years prior, she noticed a right breast mass and visited the Department of Breast and Endocrine Surgery one year prior. A hard mass 52 mm in diameter with poor mobility was found in the right breast. Mammography and breast ultrasonography revealed an irregularly shaped right breast mass with spiculated margins (Figures 1a, 1b).

A needle biopsy revealed a right invasive ductal carcinoma of the breast with fatty invasion. Immunostaining revealed estrogen receptor <1%, progesterone receptor 0%, HER2 score 3+, and a Ki-67 index of 60%. A bone radioisotope scan revealed metastasis to the 12th thoracic vertebra (data not shown). She was diagnosed with T3N3M1, stage 4 breast cancer [11]. She was treated with doxorubicin plus cyclophosphamide (AC) and trastuzumab plus docetaxel plus pertuzumab. Subsequently, bone metastasis became less clear on imaging, but the size of the breast cancer did not improve, and surgery for local control was considered. However, upon admission, she complained of palpitations, and blood assays indicated thyrotoxicosis; therefore, surgery was postponed, and she was referred to our department. She had no history of smoking or excessive alcohol consumption, and she had not been prescribed medication. Her father was diagnosed with Graves’ disease. There was no family history of breast cancer. Age at menarche was 13 years, pregnancy and childbirth were 22, 29, and 31 years, and menopause was 40 years. Her height, weight, body mass index, blood pressure, and pulse rate were 168 cm, 76 kg, 27 kg/m2, 128/71 mmHg, and 71 beats/min, respectively. The thyroid gland was elastic, soft, and enlarged, and both fingers exhibited tremors. Table 1 shows the early morning laboratory data.

**Table 1:** Early morning laboratory data recorded after admission TP: total protein; Alb: albumin; AST: aspartate aminotransferase; ALT: alanine- aminotransferase; BUN: blood urea nitrogen; Cr: creatinine; eGFR: estimated- glomerular- filtration rate; TG: triglyceride; LDL-C: low density lipoprotein cholesterol; HDL-C: high density lipoprotein cholesterol; HbA1c: glycated hemoglobin; BG: blood- glucose; WBC: white blood cell; RBC: red blood cell; Hb: hemoglobin; Plt: platelet; TSH: thyroid stimulating hormone; TRAb: thyrotrophin receptor antibody; TSAb: thyroid- stimulating antibody; TPOAb: thyroid peroxidase antibody; TgAb: thyroglobulin antibody; αSU: alpha subunit; SHBG: sex hormone- binding globulin; ACTH: adrenocorticotropic hormone; PRL: prolactin; LH: luteinizing hormone; FSH: follicle-stimulating hormone; E2: estradiol; P4: Progesterone; GH: growth hormone; IGF-1: insulin-like growth factor-1

Biochemistry	Results	Reference range	Endocrine	Results	Reference range
TP	6.7 g/dL	6.6-8.1 g/dL	TSH	1.95 µIU/mL	0.50-5.0 µIU/mL
Alb	3.8 g/dL	4.1-5.1 g/dL	Free T3	5.27 pg/mL	2.30-4.30 pg/mL
AST	28 U/L	13-30 U/L	Free T4	2.25 ng/dL	0.90-1.70 ng/dL
ALT	28 U/L	7-30 U/L	TRAb	<0.8 IU/L	<2.01 IU/L
BUN	10.6 mg/dL	8.0-20.0 mg/dL	TSAb	101%	≤120%
Cr	0.45 mg/dL	0.46-0.79 mg/dL	TPOAb	<9 IU/mL	≤28 IU/mL
eGFR	110 mL/min	≥60 mL/min	TgAb	47.1 IU/mL	≤40.0 IU/mL
TG	85 mg/dL	30-149 mg/dL	αSU	0.4 ng/mL	<1.8 ng/mL
LDL-C	120 mg/dL	65-139 mg/dL	SHBG	89.6 nmol/L	16.8-125.2 nmol/L
HDL-C	61 mg/dL	40-103 mg/dL	ACTH	14.8 pg/mL	7.2-63.3 pg/mL
HbA1c	5.3%	4.9-6.0%	Cortisol	5.5 µg/dL	3.7-19.4 µg/dL
BG	110 mg/dL	73-109 mg/dL	PRL	7.17 ng/mL	3.10-15.40 ng/mL
			LH	43.1 mIU/mL	11.0-50.0 mIU/mL
Hematology			FSH	69.6 mIU/mL	26.0-120.0 mIU/mL
WBC	5350 /µL	3300-8600 /µL	E2	17.5 pg/mL	≤47.0 pg/mL
RBC	438×10^4^ /µL	386×10^4^-492×10^4^ /µL	P4	0.16 ng/mL	0.0-0.30 ng/mL
Hb	14.1 g/dL	11.6-14.8 g/dL	GH	1.32 ng/mL	0.13-9.88 ng/mL
Plt	18.2×10^4^ /μL	15.8×10^4^-34.8×10^4^ /μL	IGF-1	79 ng/mL	78-213 ng/mL

**Figure 2:**
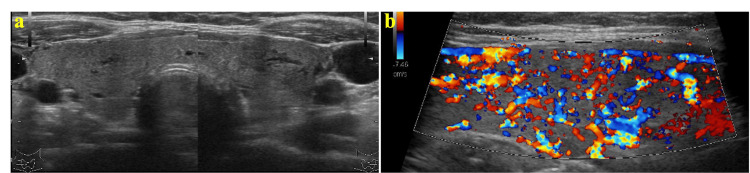
Thyroid echo images a: The total view of the thyroid gland. b: The blood flow within the thyroid gland.

When the assay kit was changed (TOSOH CORPORATION) and retested, the thyroid function remained unchanged. Thyrotrophin receptor and thyroid-stimulating antibody tests were negative, whereas a thyroglobulin antibody test was positive. The alpha subunit was 0.4 ng/mL, and the sex hormone-binding globulin (SHBG) was 89.6 nmol/L. No genetic mutations in thyroid hormone receptor beta were found. Thyroid echocardiography revealed an enlarged isthmus and bilateral lobes, a heterogeneous interior, and increased blood flow (Figures 2a, 2b).

**Figure 3:**
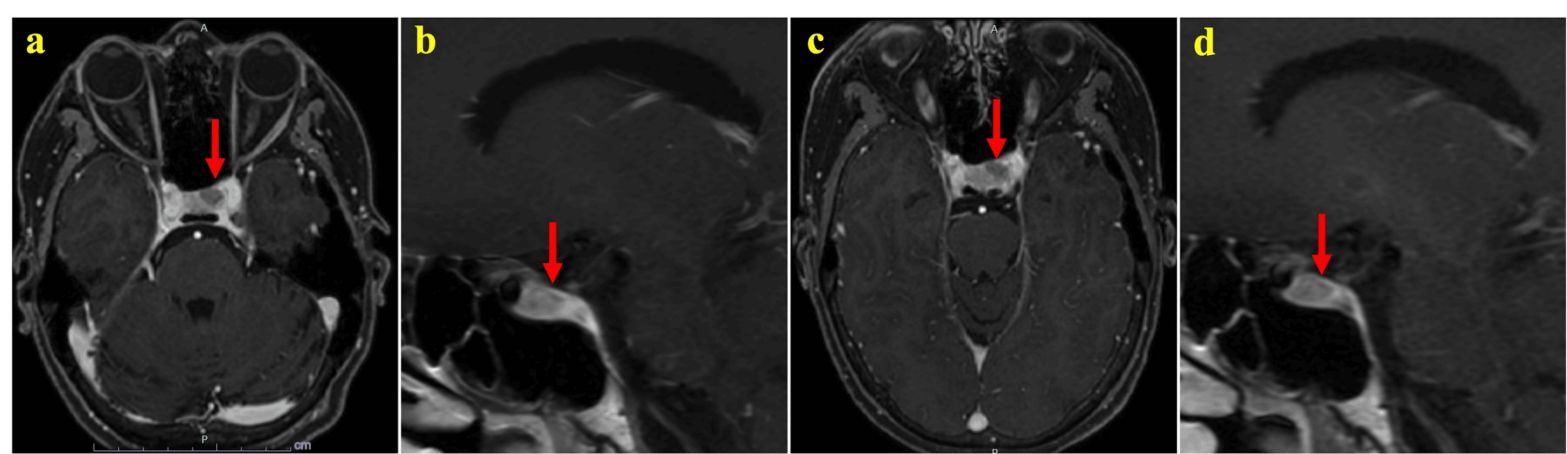
Pituitary magnetic resonance imaging Contrast-enhanced images are shown. a and b: Axial and sagittal views, respectively, taken on admission, show a 6 mm-diameter nodule with poor contrast effect (arrows). c and d: axial and sagittal views, respectively, taken seven months after the initiation of lanreotide treatment and are similar in size to a and b (arrows).

Scintigraphy of the thyroid gland with technetium showed a diffusely increased uptake rate, with a right lobe uptake rate of 3.5% and a left lobe uptake rate of 2.7% (data not shown). Contrast-enhanced magnetic resonance imaging (MRI) of the pituitary gland showed a 6-mm-diameter nodule with a poor contrast effect in the pituitary gland, which was not markedly different from that observed 12 years earlier (Figures 3a, 3b).

A thyrotropin-releasing hormone load test showed no TSH response (Table 2), and the corticotropin-releasing hormone and luteinizing hormone-releasing hormone load tests showed no abnormal secretion of other pituitary hormones. In the octreotide suppression test, TSH of 2.44 μIU/mL before loading was reduced to 1.5 μIU/mL eight hours after loading, but not in the bromocriptine suppression test (data not shown).

**Table 2:** Thyrotropin-releasing hormone load test TSH: thyroid stimulating hormone

	Before load	After 30 minutes	After 60 minutes	After 90 minutes	After 120 minutes
TSH (μIU/mL)	2.31	2.85	2.62	2.44	2.48

**Figure 4:**
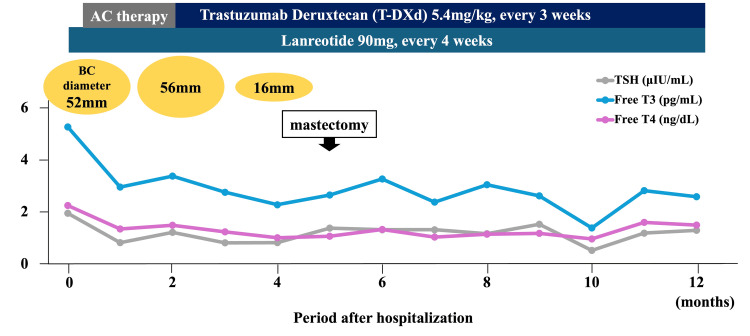
Clinical course after hospitalization The gray line shows the TSH level. The blue line shows the Free T3 level. The pink line shows the Free T4 level. AC therapy: doxorubicin plus cyclophosphamide therapy; BC: breast cancer

Therefore, she was clinically diagnosed with TSH-PiTNET. Her clinical course is shown in Figure 4.

We aimed to normalize pituitary and thyroid functions with lanreotide, which occurred after one month of treatment. The breast cancer diameter increased to 56 mm, and the AC therapy was switched to T-DXd. Four months later, the breast cancer diameter was remarkably reduced to 16 mm in diameter, and locoregional surgery was performed five months later. Histological examination of the excised specimen revealed no malignant cells, and she attained a pathologically complete response. Post-operative chemotherapy was continued to prevent recurrence and metastasis. A pituitary MRI performed seven months after the initiation of lanreotide showed no significant nodal changes (Figures 3c, 3d). Surgery was considered for pituitary tumors after the completion of chemotherapy. 

**Figure 5:**
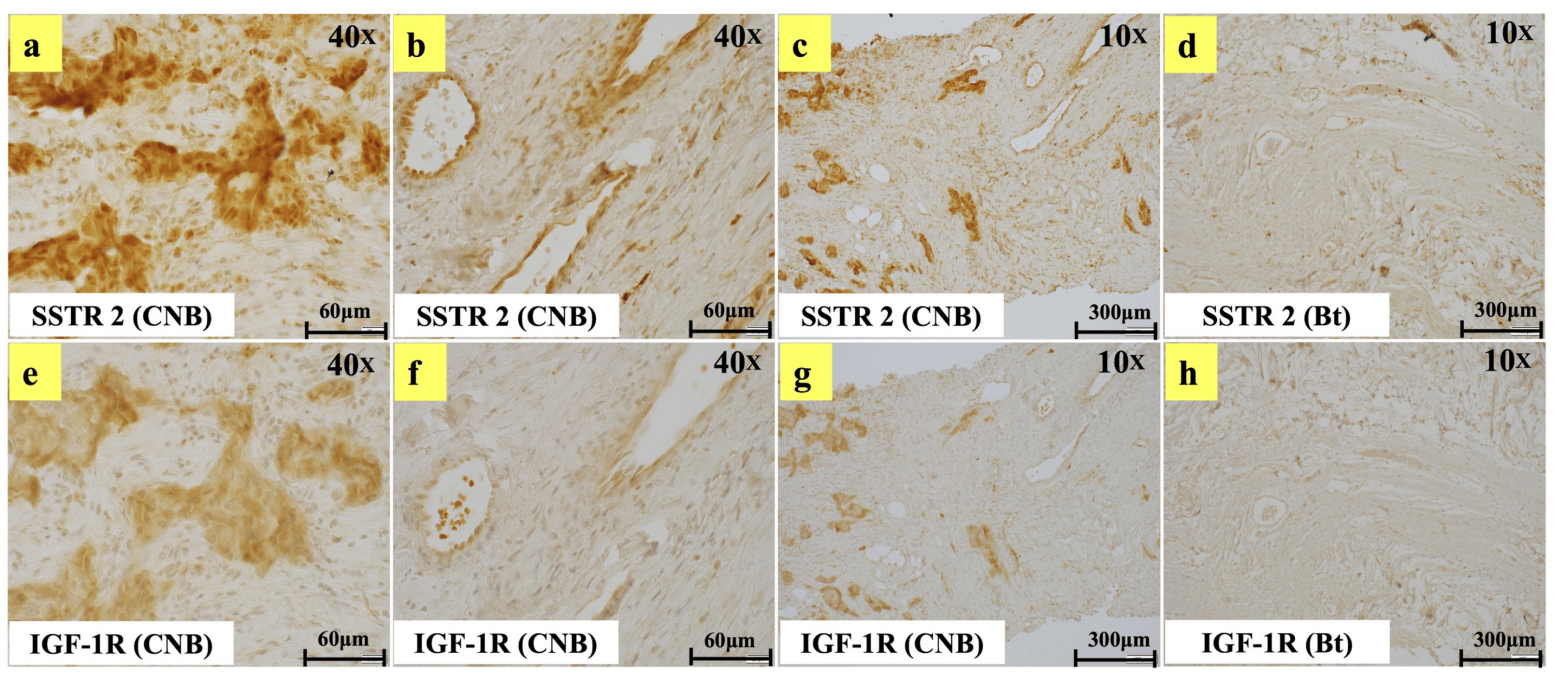
Immunostaining image Images of breast cancer needle biopsy and mastectomy specimens immunostained for SSTR2 and IGF-1R are shown. a, b, and c: Breast cancer needle biopsy specimens were immunostained for SSTR2. d: Mastectomy specimen (without cancer cells) immunostained for SSTR2. e, f, and g: Breast cancer needle biopsy specimens immunostained for IGF-1R. h: Mastectomy specimen (without cancer cells) immunostained for IGF-1R. a and e show cancer cells, and b and f show blood vessels at 40x magnification. c, d, g, and h are 10x magnifications showing larger areas. SSTR2: somatostatin receptor 2; IGF-1R: insulin-like growth factor 1 receptor; CNB: core needle biopsy; Bt: total mastectomy

Paraffin blocks were prepared from the needle biopsy specimens of the breast cancer and mastectomy specimens, and 4-μm-thick paraffin sections were prepared. After deparaffinization with xylene, antigen activation (heat treatment with antigen retrieval DAKO (PH9) at 120 °C for 20 minutes) was performed. After blocking (G-Block, 30 min at room temperature), the primary antibody (4°C, overnight) was added. Next, the secondary antibody (room temperature, 60 min) was reacted with. Finally, the cells were stained with Dako 3,3' Diaminobenzidine. The primary antibodies used and their dilutions were as follows: anti-SSTR2 antibody (Proteintec Group, Inc., 50x dilution) and anti-IGF-1R antibody (Proteintec Group, Inc., 100x dilution). The secondary antibody used was Dako Envision Dual Link System-horseradish peroxidase (HRP). The results of immunostaining are shown in Figure 5: both SSTR2 and IGF-1R appeared diversely in the plasma membrane and cytoplasm of tumor cells (Figures 5a, 5e). Immunoreactivity was observed not only in tumor cells but also in peritumoral structures (blood vessels and stromal cells) (Figures 5b, 5f). Mastectomy specimens were not stained (Figures 5d, 5h).

## Discussion

This case study has three unique aspects. First, this is the first report of TSH-PitNET combined with breast cancer; second, the breast cancer specimen was positive for SSTR2 and IGF-1R immunoreactivity; third, the breast cancer showed a complete response after treatment with SSA and chemotherapy.

Based on their clinical course, TSH-PitNETs are thought to develop first, followed by breast cancer. An association between excess thyroid hormones and breast cancer has been reported [1]. Thyroid hormones increase the estrogen/androgen ratio by two main mechanisms. First, they directly stimulate peripheral aromatase, which in turn produces estrogen. Secondly, it increases hepatic SHBG synthesis, which stimulates peripheral aromatase [2]. The present patient was exposed to slightly elevated thyroid hormone levels for a long period due to chronic TSH stimulation, which could have put her at risk of breast cancer.

Somatostatin is a cyclopeptide that inhibits both hormone secretion and neuronal excitability. Its physiological function is mediated by five G protein-coupled receptors called SSTR1-5. These five receptors regulate receptor-specific hormones, the secretory neurotransmission of growth factors, and cell proliferation. SSTR1, 2, 4, and 5 induce cell cycle arrest, whereas SSTR3 induces apoptosis [12]. Thus, it is interesting that SSTRs are expressed in malignant tumors, even though SSTRs exhibit antitumor effects. In fact, the expression of SSTR2 has been reported in approximately 50-90% of breast cancers [5,6] but has not been confirmed in normal breast tissue [5,13]. The fact that SSTRs are expressed during malignant transformation may be a normal response of the body to resist and repair cancer cells. One study reported that the overexpression of SSTR2 induces apoptosis in breast cancer cells, decreases epidermal growth factor receptor expression, and exhibits antiproliferative effects [14].

IGF-1 is composed of four domains and is produced in the liver by the direct action of growth hormone (GH). IGF-1R is a 7.7-kDa single-chain polypeptide encoded on chromosome 12. In contrast to SSTR2, IGF-1R has been implicated in tumor growth, invasion, and metastasis, and the binding of IGF-1 and IGF-1R triggers a proliferative and anti-apoptotic signaling cascade [15,16]. The phosphorylation of IGF-1R activates the phosphatidylinositol 3-kinase/AKT kinase and rapidly accelerated fibrosarcoma kinase/mitogen-activated protein kinase (RAF/MAPK) pathways, leading to tumor progression [7].

Interestingly, in the present case, both SSTR2 and IGF-1R appeared on the plasma membrane and in the cytoplasm, showing immunoreactivity not only in tumor cells but also in peritumoral structures (blood vessels and stromal cells). This is consistent with the results of previous reports [7,8].

In a domestic open-label Phase III study showing the efficacy and safety of SSAs for TSH-PitNET, patients were treated with 60-120 mg/week of lanreotide, and normalization of TSH, freeT3, and freeT4 was observed in 51.8%, 76.9%, and 69.2% of patients, respectively, at four weeks post-dose and was maintained for 48 weeks [17]. In the present case, the pituitary and thyroid functions normalized after one month of lanreotide administration and remained normal throughout the course of treatment. The DESTINY-Breast03 trial, which compared T-DXd with trastuzumab-emtasine therapy, reported a 21% complete response rate for T-DXd in breast cancer [18]. In this case, it is difficult to assert that lanreotide caused a reduction of breast cancer cells because of the close timing of the initiation of T-DXd and lanreotide. However, given the mechanism of action of lanreotide, it is possible that combination treatment with chemotherapy and lanreotide had a positive effect on breast cancer. The general mechanisms underlying the antitumor effects of SSAs are as follows [9,19,20]. Direct mechanisms include 1) phosphotyrosine phosphatase activation, 2) tyrosine kinase inhibition, 3) cell cycle arrest by downregulation of phosphorylation and activation of the rat sarcoma virus/MAPK pathway, 4) apoptosis of cancer cells by intracellular acidification and endonuclease activation, and 5) inhibition of GH and cytokines by inhibition of cyclic adenosine monophosphate and calcium production. Indirect mechanisms include 1) direct inhibition of IGF-1 gene expression or GH-dependent IGF-1 synthesis in the liver, 2) immunomodulatory effects: inhibition of lymphocyte proliferation, immunoglobulin synthesis, and cluster of differentiation 4+ (CD4+) T cell-derived interferon-γ synthesis, and 3) inhibition of tumor nutrient supply through peritumoral vasoconstriction. However, reports of clinical trials of SSAs in breast cancer patients have not always been effective [5,9,10], probably for the following reasons [9]: 1) heterogeneous expression of SSTRs in tumor tissue, 2) differences in affinity for SSAs among different types of SSTRs, 3) the doses of SSAs in animal models are higher than in humans, and 4) many patients in clinical trials were not evaluated for SSTRs. Since the anti-proliferative effect of SSAs depends on the presence of SSTRs, it is critical to confirm the expression pattern of SSTRs in tumor cells. SSAs have the advantage of long-term treatment with fewer side effects and without compromising quality of life. The combination of SSAs and chemotherapy for patients with breast cancer with positive SSTR2 expression may be a new treatment to achieve synergistic antitumor effects.

## Conclusions

We report the case of a patient with SSTR2-positive breast cancer and TSH-PitNET who, after treatment with T-DXd and lanreotide, showed marked shrinkage of the breast mass, disappearance of cancer cells, and normalization of pituitary and thyroid functions. Confirmation of the expression pattern of SSTRs in tumor cells could lead to the effective use of SSAs and the development of new therapeutic agents. Further studies are needed to prove the efficacy of SSAs in SSTR-positive breast cancer patients. Since SSAs act on all aspects of hormone secretion and have antitumor effects, it is important to choose a treatment regimen that considers their effects on other diseases and their interactions with other drugs.
